# Interaction of THP-1 Monocytes with Conidia and Hyphae of Different *Curvularia* Strains

**DOI:** 10.3389/fimmu.2017.01369

**Published:** 2017-10-18

**Authors:** Eszter Judit Tóth, Éva Boros, Alexandra Hoffmann, Csilla Szebenyi, Mónika Homa, Gábor Nagy, Csaba Vágvölgyi, István Nagy, Tamás Papp

**Affiliations:** ^1^MTA-SZTE Fungal Pathogenicity Mechanisms Research Group, Hungarian Academy of Sciences, University of Szeged, Szeged, Hungary; ^2^Faculty of Science and Informatics, Department of Microbiology, University of Szeged, Szeged, Hungary; ^3^Hungarian Academy of Sciences, Biological Research Centre, Szeged, Hungary

**Keywords:** *Curvularia*, monocyte, invasive mycosis, melanin, quantitative reverse transcription PCR, ELISA, interleukin-10

## Abstract

Interaction of the human monocytic cell line, THP-1 with clinical isolates of three *Curvularia* species were examined. Members of this filamentous fungal genus can cause deep mycoses emerging in both immunocompromised and immunocompetent patients. It was found that monocytes reacted only to the hyphal form of *Curvularia lunata*. Cells attached to the germ tubes and hyphae and production of elevated levels of interleukin (IL)-8 and IL-10 and a low level of TNF-α were measured. At the same time, monocytes failed to produce IL-6. This monocytic response, especially with the induction of the anti-inflammatory IL-10, correlates well to the observation that *C. lunata* frequently cause chronic infections even in immunocompetent persons. Despite the attachment to the hyphae, monocytes could not reduce the viability of the fungus and the significant decrease in the relative transcript level of HLA-DRA assumes the lack of antigen presentation of the fungus by this cell type. *C. spicifera* and *C. hawaiiensis* failed to induce the gathering of the cells or the production of any analyzed cytokines. Monocytes did not recognize conidia of *Curvularia* species, even when melanin was lacking in their cell wall.

## Introduction

Members of the ascomycete genus, *Curvularia* includes primarily saprotrophic and plant pathogenic filamentous fungi. Some of them, such as *Curvularia lunata, C. hawaiiensis*, and *C. spicifera*, however, are also considered as emerging agents of local and invasive human phaeohyphomycoses (1), i.e., infections caused by melanin producing molds. *Curvularia* species are frequently reported as agents of allergic fungal sinusitis and bronchopulmonary disease (2–4) but they can also be associated with mycotic keratitis (5, 6), cutaneous and subcutaneous mycoses (7, 8), and infections of the central nervous system (2, 9). Deep and disseminated *Curvularia* infections have been described in both immunocompromised and immunocompetent patients (4, 10–13). Increasing prevalence of these infections has been reported during recent years (4, 14, 15). Despite that *Curvularia* species are among the most common dematiaceous fungi isolated from clinical samples (16), experimental data concerning the background of their pathogenicity and interactions with the host are very limited (2).

Human monocytes are circulating cells of innate immunity that can further differentiate into macrophages or dendritic cells and capable of phagocytoses, cytokine production, and antigen presentation. They play a pivotal role in the host response to fungal infections and their activity against the conidia, germlings, and hyphae of *Aspergillus fumigatus* was proven and analyzed previously (17, 18).

THP-1 is a human monocytic cell line isolated from a patient with acute monocytic leukemia. This cell line can be differentiated into macrophages and are widely used to study monocyte and macrophage functions and in immune modulation studies (19). For example, THP-1 monocytes treated with LPS, showed altered expression of several inflammation related genes, such as *IL1B, IL6, IL8, IL10*, and *TNFA* (19, 20). Several studies have compared the responses of THP-1 monocytes and human peripheral blood mononuclear cell derived monocytes to a variety of stimuli and the two cell types showed relatively similar response patterns in most cases (19). THP-1 monocytes and macrophages has also been used successfully as *in vitro* models to examine host–pathogen interactions for various fungal agents, such as *A. fumigatus* (18, 21–23), *Candida albicans* (24), *Candida glabrata* (25), and *Cryptococcus neoformans* (26).

In this study, interactions of THP-1 monocytes with conidia and hyphae of three fungal strains representing *C. hawaiiensis, C. lunata*, and *C. spicifera* were analyzed to get a first insight into the host response to *Curvularia* species. For comparison, an *A. fumigatus* strain from a clinical origin was also involved in the study.

## Materials and Methods

### Fungal Strains, Culture Conditions, and Inoculum Preparation

*Curvularia lunata* SZMC 23759 and *C. spicifera* SZMC 13064, *A. fumigatus* SZMC 23245 isolated from human eye infections and *C. hawaiiensis* CBS 103.97 isolated from human sinusitis with ophthalmic and cerebral involvement were used in the study. Strains were grown on potato dextrose agar (PDA; VWR International) at room temperature. To block melanin synthesis, PDA was supplemented with 20 µg/ml tricyclazole (Sigma-Aldrich) and 1% of agar (Merck). For the interaction studies, conidial suspensions (10^5^ conidia/ml) were prepared in phosphate buffer saline (PBS; 137 mM NaCl, 2.7 mM KCl, 10 mM Na_2_HPO_4_, 2 mM KH_2_PO_4_, pH 7.4) by washing the conidia from 14 and 7 days old fungal cultures in case of the *Curvularia* strains and the *Aspergillus* strain, respectively. To get rid of hyphal debris, spore suspensions were filtered through a filter paper with a pore size of 45 µm (Millipore). Heat inactivation of the conidia was performed at 125°C for 25 min.

### Culturing and Infection of the THP-1 Cells

THP-1 cells were maintained in RPMI 1640 medium (Gibco) supplemented with 10% (v/v) heat inactivated fetal bovine serum (FBS; Gibco) and 1% (v/v) antibiotic/antimycotic solution containing 10,000 U/ml of penicillin, 10,000 µg/ml of streptomycin and 25 µg/ml of Amphotericin B (Gibco) at 37°C in a humidified incubator with 5% CO_2_. For interaction studies, THP-1 cells (10^5^ cells/ml) were placed on 6-well or 12-well cell culture plates with flat bottoms (Sarstedt) in 3 or 1 ml RPMI 1640 medium supplemented with 10% heat inactivated FBS and without antibiotic the day before infection, respectively.

Number of the *Curvularia* conidia and cells were set to maintain an effector (THP-1) to target (conidia) (E:T) ratio of 20:1, while for *A. fumigatus* E:T ratio was 20:1 or 1:2. Because of the small size of the conidia of *A. fumigatus*, an E:T ratio of 1:2 proved to be optimal for this fungus in agreement with the literature data (18). For the *Curvularia* strains, we tested various E:T ratios and that of 20:1 proved to be applicable, mainly because of the large size and the relatively short germination time (approx. 1.5 h) of their conidia. Cells were incubated with or without the fungi at 37°C in a humidified incubator with 5% CO_2_ for 3, 9, or 24 h. When monocytes were treated with lipopolysaccharide (LPS, *Escherichia coli* Q26:B6; Sigma-Aldrich), 1 µg/ml final concentration of LPS was used. Microscopic examination of the interactions was performed using a Leica DMI4000 B inverse microscope (Leica Microsystems). All experiments were performed in two technical and three biological replicates.

### Phagocytosis Assay

THP-1 cells (10^5^ cells/ml) were seated on a 12-well plate one day before the experiment. Four hours before the assay, cells were stained with CellMask Deep Red Plasma Membrane stain (Thermo Scientific) in a 0.5-fold concentration for 15 min and washed twice with PBS. Conidia were stained with AlexaFluor 488 carboxylic acid, succinimidyl ester (Thermo Scientific) for 15 min and washed twice with PBS at 4°C to prevent germination. The E:T ratio was 1:2 or 20:1. For analysis, collected samples were centrifuged with 1,000 rpm for 15 min and resuspended in 200 µl PBS supplemented with 0.05% Tween-20 (Reanal). Interaction and phagocytosis were measured after 1 or 3 h using a FlowSight Imaging Flow Cytometer (Amnis) and evaluated with the IDEAS Software (Amnis).

### Real-time Quantitative Reverse Transcription PCR (qRT-PCR) Analysis

Total RNA was extracted from the THP-1 cells using the RNeasy Mini kit (Qiagen) according to the instructions of the manufacturer. The final elution volume was 20 µl. cDNA was synthesized using the SuperScript VILO Master Mix (Invitrogen) following the protocol of the manufacturer. qRT-PCR was carried out using StepOne Plus Real-Time PCR System (Applied Biosystems). Reactions were performed by either the TaqMan Gene expression Master Mix (Applied Biosystems) or the Sybr Select Master Mix (Applied Biosystems) using the probes and primers listed in Tables 1 and 2, respectively, according to the manufacturer’s protocols. Relative transcript levels were calculated with the ΔΔC_T_ (2^−ΔΔCt^) method (27) using a fragment of the 18S rRNA coding gene for normalization. All measurements were performed in two technical and three biological replicates.

**Table 1:** TaqMan probe details (Applied Biosystems) used in the qRT-PCR analyses.

Target gene	TaqMan assay ID
18S rDNA	Hs99999901_s1
*IL10*	Hs00961522_m1
*IL6*	Hs00174131_m1
*IL8*	Hs00174103_m1
*TNFA*	Hs00174128_m1
*NLRC3*	Hs01054716_m1

**Table 2:** Primer pairs used in the qRT-PCR analyses.

Target gene	Forward (5′–3′) primer	Reverse (5′–3′) primer	Product length (bp)
*CCR1*	TCTACGCCTTCGTTGGTGAG	TTTAACCAGGTGCACAGCCA	80
*CCR2*	TACCAACGAGAGCGGTGAAG	GCATGTTGCCCACAAAACCA	149
*CCR5*	TTACTGTCCCCTTCTGGGCT	AAGCAAACACAGCATGGACG	168
*CXCR2*	TTTCGCCATGGACTCCTCAA	GTAGTGGAAGTGTGCCCTGA	116
*IL1B*	AGCTGGAGAGTGTAGATCCCAA	GGGAACTGGGCAGACTCAAA	112
*ITGAL*	GCAAGGACATACCGCCCAT	TACTCAGGCTCAGCTCCACA	186
*ITGAM*	AGTCTGCCTCCATGTCCAGAA	CTGCGTGTGCTGTTCTTTGTC	144
*ITGAX*	AGTCTGCCTCCATGTCCAGAA	CTGCGTGTGCTGTTCTTTGTC	103
*HLADRA*	CCGATCACCAATGTACCTCCA	CGAAGCCACGTGACATTGAC	128

### Cytokine Assays

To confirm the cytokine concentrations in the culture supernatants, DuoSet ELISA Kits (R&D Systems) were used for tumor necrosis factor alpha (TNF-α), interleukin (IL)-6, and IL-8 according to the instructions of the manufacturer. To measure the IL-10 level, the Human IL-10 ELISA kit (Immunotools) was used. Cytokine titers were calculated by reference to standard curves generated by the four parameters logistic curve-fit method.

### Viability Test

To measure the hyphal damage after the incubation with monocytes, a colorimetric assay using 3-(4,5-dimethylthiazol-2-yl)-2,5-diphenyltetrazolium bromide (MTT; Sigma-Aldrich) was performed (28) on 12-well culture plates. After incubation for 3, 6, or 24 h, monocytes were lysed with 0.5% sodium deoxycholate (Sigma-Aldrich) and the wells were washed three times with PBS. Then, 1 ml RPMI 1640 supplemented with 1% MTT was added to the wells and the plates were incubated at 37°C, under 5% CO_2_ concentration for 3 h. After removing the supernatant, wells were washed two times with PBS and stored at −20°C overnight. Before detection, 1 ml acidic isopropanol (95% isopropanol and 5% 1 N HCl) was added into the wells and the plates were incubated until the blue color dissolved from hyphae. Absorbance of the supernatant was measured at the wavelength of 550 nm using a spectrophotometer (Spectrostar Nano, BMG Labtech). Viability of the hyphae was calculated using the following formula, where *OD*_550_ sample was the absorbance measured for the hyphae incubated with monocytes and *OD*_550_ control was the absorbance measured for the hyphae incubated without monocytes:$\text{Viability}=\left(\frac{\mathrm{OD}550\;\text{sample}}{\mathrm{OD}550\;\text{control}}\right)\times 100.$

### Statistical Analysis

All measurements were performed in at least two technical and three biological replicates. Significance was calculated with paired *t*-test using Microsoft Excel of the Microsoft Office package. *P* values less than 0.05 were considered statistically significant.

## Results

### THP-1 Cells Do Not Phagocytose *Curvularia* spp. Conidia and Hyphae

**Figure 1:**
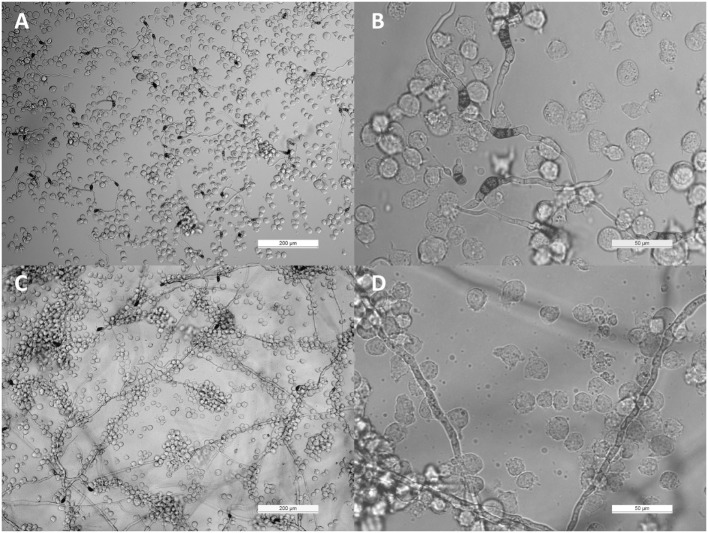
Interaction of THP-1 monocytes with *Curvularia lunata*. Light micrographs were taken at 3 [panels **(A,B)**] and 24 h [panels **(C,D)**] postinoculation; the E:T ratio was 20:1.

**Figure 2:**
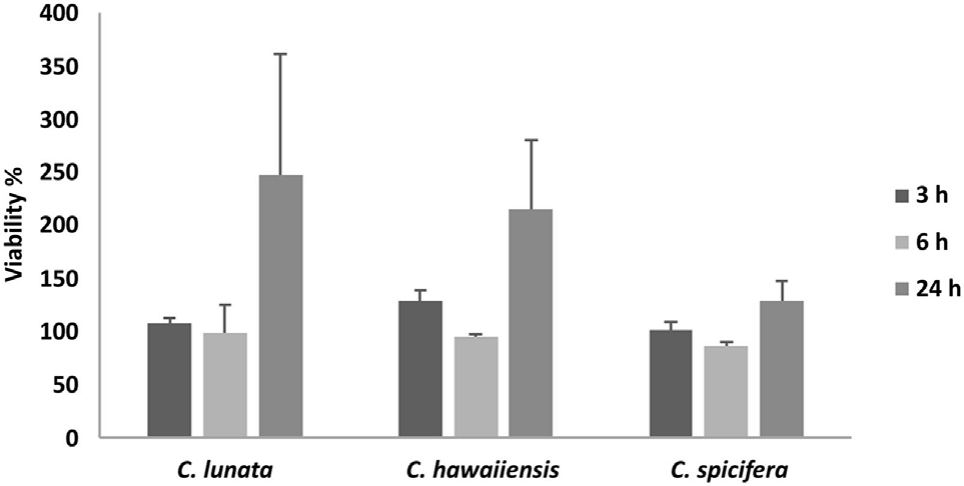
Cell viability (MTT) assay of the three *Curvularia* strains incubated together with THP-1 monocytes for 3 and 24 h. Results are presented as averages of three independent experiments; error bars represent SDs.

THP-1 cells were confronted with *C. lunata, C. hawaiiensis*, and *C. spicifera* by co-incubating heat inactivated or living conidia with the monocytes. Interactions were analyzed after 3 and 24 h of confrontation. In case of the living conidia, germination was already in progress and germ tubes were developed at 3 h postinoculation (Figures 1A,B) while branching hyphae were present at 24 h postinoculation (Figures 1A,B). Microscopic analysis revealed that the monocytes were not able to phagocytose the conidia of any tested strains; moreover, they did not attract to them at all (Figures 1A,B). The absence of the monocytic response did not depend on the melanin content as it also missed in case of those conidia of *C. lunata* where the melanin biosynthesis had been previously blocked. At the same time, THP-1 cells aggregated around and attached to the hyphae of *C. lunata* (Figures 1C,D). However, monocytes did not block the germination of the conidia and MTT assay did not detect hyphal damage in the tested *Curvularia* strains (Figure 2). For comparison, MTT assay was also performed with *A. fumigatus* under the same co-incubation conditions. In this case, viability of the hyphae decreased to 12.9% after 24 h of interaction with the THP-1 cells.

**Figure 3:**
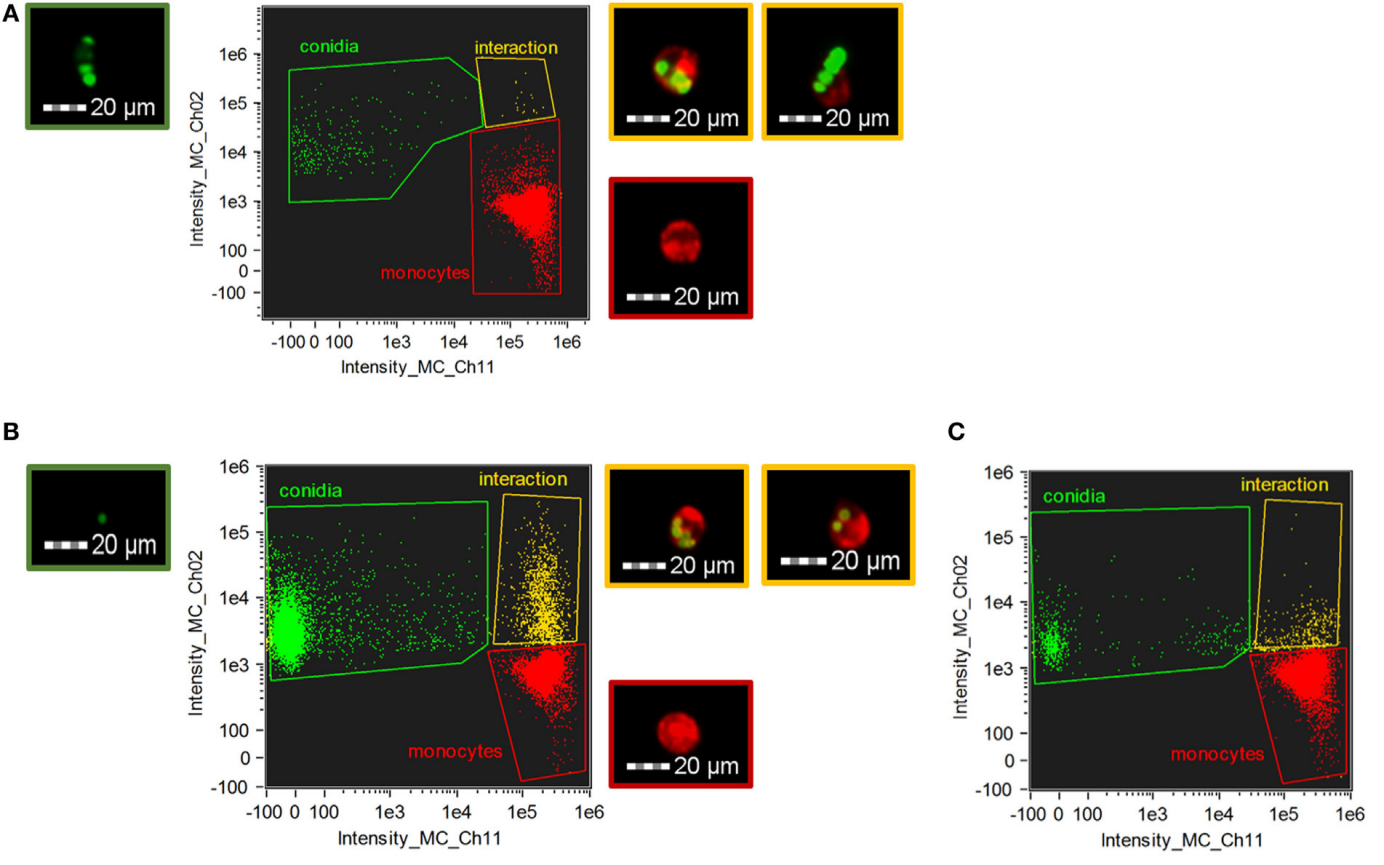
Phagocytosis of *Curvularia lunata* **(A)** and *Aspergillus fumigatus* **(B,C)** conidia by THP-1 monocytes. THP-1 cells and conidia were stained with CellMask Deep Red Plasma Membrane Stain and Alexa Fluor 488 carboxylic acid, succinimidyl ester, respectively. Number of the *Curvularia* conidia and cells were set to maintain an E:T ratio of 20:1 **(A)**, while for *A. fumigatus* E:T ratio was 1:2 **(B)** or 20:1 **(C)**. Monocytes were identified by detecting fluorescence intensity on channel 11 (Intensity_MC_CH_11) while channel 2 (Intensity_MC_CH_2) was used to detect the conidia. Cells and conidia were co-incubated for 1 h. Fluorescent micrographs showing conidia (green border) and THP-1 cells alone (red border) and in interaction (i.e., phagocytosis or attachment) (yellow border) were recorded during the imaging flow cytometry.

For quantification and a more detailed analysis, phagocytosis was also examined by imaging flow cytometry in case of *C. lunata* and *A. fumigatus* (Figure 3). THP-1 cells actively phagocytosed the living conidia of *A. fumigatus* already at 1 h after their confrontation; the mean ratio of the phagocyting cells counted from three biological replicates was found to be 2.4 (±0.4)% and 20.7 (±2.5)% at E:T ratios of 20:1 (Figure 3C) and 1:2 (Figure 3B), respectively. At the same time, the number of interacting cells and conidia proved to be insignificant in the case of *C. lunata*. Only 0.12 (±0.1)% of the monocytes were attached to or ingesting the conidia at 1 h after the start of the interaction (the E:T ratio was 20:1). Similar values were measured for heat inactivated and melanin blocked conidia, 0.35 (±0.2)% and 0.69 (±0.1)% after 1 h of interaction, respectively. Image analysis revealed that majority of these small numbers of interactions detected by flow cytometry was only attachment of the monocytes to the conidia instead of real phagocytosis (Figure 3A).

### qRT-PCR Analysis of Immune-Relevant Genes Induced by Germinating Conidia and Hyphae of *Curvularia* Species

**Figure 4:**
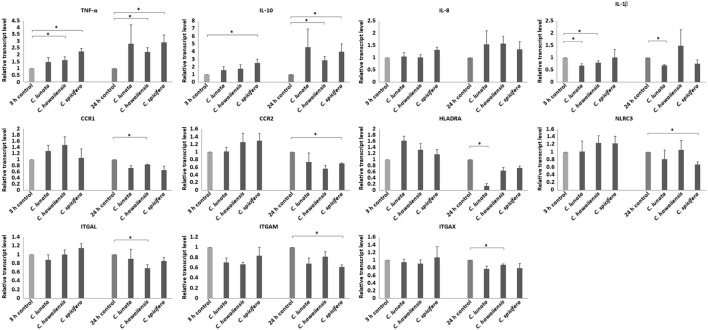
Relative transcript levels of pro- and anti-inflammatory cytokines, activation and antigen presentation related genes. THP-1 monocytes were confronted with *Curvularia* strains for 3 and 24 h. After total RNA extraction from the monocytes, cDNA synthesis, and quantitative reverse transcription PCR were carried out. Relative transcript levels were calculated by the 2^−ΔΔCt^ method. Presented values are averages of the results of three independent experiments; error bars represent SDs. Relative transcript values followed by * significantly differed from the control (taken as 1), according to the paired *t*-test (*p* < 0.05).

Three and 24 h after the infection of THP-1 monocytes with conidia, cells were harvested and the relative transcript level of certain activation related and cytokine or chemokine coding genes were measured. Significant changes were not detected in the transcription of the genes encoding the chemokine receptors CCR5 (C-C chemokine receptor type 5), CXCR2 (IL-8 receptor, beta) and the cytokine IL-6 at both tested times in any interactions. Decreased relative transcript levels were detected for the genes of the chemokine receptors CCR1 (C-C chemokine receptor type 1) and CCR2 (C-C chemokine receptor type 2) after 24-h interaction with the tested *Curvularia* strains (Figure 4). Similarly, slightly decreased transcript levels were measured for the genes encoding the adhesion molecules ITGAL (integrin subunit alpha L), ITGAM (integrin subunit alpha M) and ITGAX (integrin subunit alpha X) after 24 h of co-incubation. In case of the gene encoding IL-1β, NLRC3 (NLR family CARD domain containing 3) either no significant changes in the transcription or decreased transcript levels were detected after 24 h of interaction (Figure 4). Transcription of gene of HLA-DRA (HLA class II histocompatibility antigen, DR alpha chain) showed significant reduction in case of *C. lunata*. Increased transcription of the gene encoding TNF-α was observed in response to all tested strains after both 3 and 24 h (Figure 4). A trend for increased transcript levels was detected for the genes of IL-8 and IL-10 but significant changes were detected only in interactions with certain strains (i.e., with *C. spicifera* for IL-8 and with *C. hawaiiensis* and *C. spicifera* for IL-10) (Figure 4).

In case of *A. fumigatus*, transcript levels were measured after 3, 9, and 24 h of interaction, because this fungus starts to germinate at about 7 h after inoculation. In accordance with the literature data (18), conidia did not induce the transcription of *IL6, IL8, IL10*, and *TNFA* while significantly increased relative transcript levels were measured for *TNFA* after 9 h of interaction (67.66 ± 10.9) and *IL8* at 9 (8.86 ± 0.73) and 24 h (24.03 ± 2.93) (see Figure S1 in Supplementary Material).

As control, THP-1 cells were also stimulated with LPS and the relative transcript levels of *IL1b, IL6, IL8, IL10, TNFA, CCR1*, and *CCR2* were measured as above. As expected, all tested genes showed significantly altered expression after LPS treatment. Expression of *IL1b, IL6, IL8, IL10*, and *TNFA* was induced showing significantly increased relative transcript levels compared to the untreated control, in contrast, relative transcript levels of *CCR1* and *CCR2* were found to be significantly decreased (see Figure S2 in Supplementary Material).

### IL-6, IL-8, IL-10, and TNF-α Production in Response to Germinating Conidia and Hyphae of *Curvularia* Species

**Figure 5:**
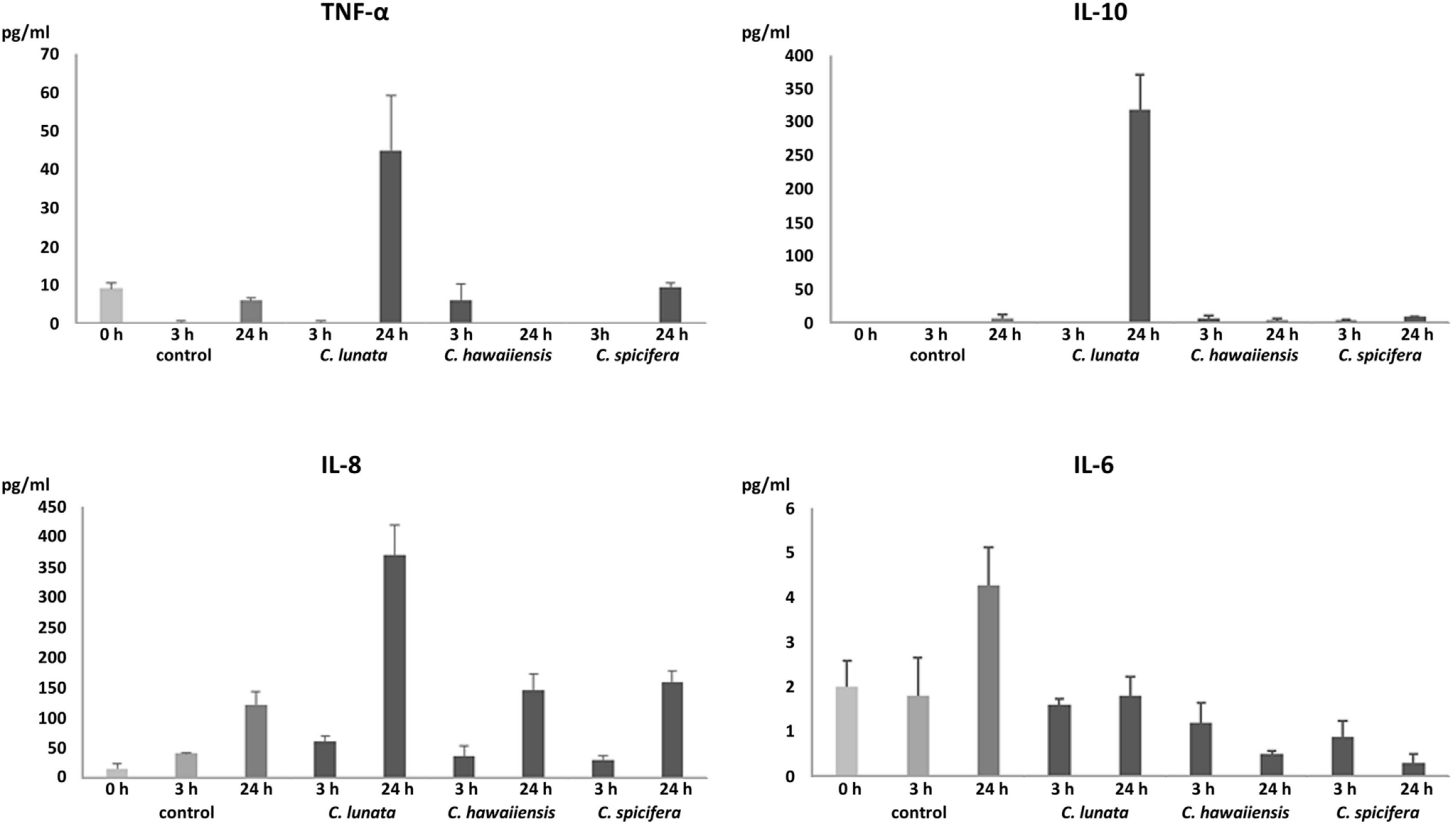
Quantity of produced pro- and anti-inflammatory cytokines (pg/ml) after interaction with *Curvularia* strains for 3 and 24 h. THP-1 cells were interacted with the fungal conidia in an E:T ratio of 20:1. In case of the control, monocytes were incubated without the fungi. Concentrations of IL-6, IL-8, IL-10, and TNF-α in the culture supernatant were measured by ELISA. Cytokine titers were calculated by reference to standard curves generated by the four parameters logistic curve-fit method. Results are presented as averages from three independent experiments; error bars represent SDs.

In agreement with the results of the transcription analysis, no significant IL-6 production was observed in all interactions (Figure 5). In response to *C. lunata*, THP-1 cells showed significant IL-8 (340 pg/ml) and IL-10 (318 pg/ml) production after 24 h of interaction (Figure 5). Confronting with *A. fumigatus*, monocytes displayed similar IL-8 production (376 pg/ml) but only a low amount of IL-10 (43 pg/ml) could be detected. Infection with the other two *Curvularia* strains did not cause significant change in the IL-8 and IL-10 level. In case of *C. lunata*, a moderate increase in the TNF-α level (45 pg/ml) was also measured at 24 h postinoculation (Figure 5).

### Response of THP-1 Cells to the Conidia of *C. lunata*

Response of monocytes to non-germinating and germinating conidia were compared in case of *C. lunata*. In this experiment, activation of cytokine and chemotactic genes and cytokine production was analyzed by qRT-PCR and ELISA assays, respectively, after interactions of THP-1 cells with heat inactivated conidia (see Figures S3 and S4 in Supplementary Material). As melanin content may hamper the monocyte response, non-melanized and heat inactivated conidia, which were harvested after blocking the melanin biosynthesis during cultivation, were also tested. Compared to the germinating conidia and hyphae (Figures 4 and 5), presence of heat inactivated conidia, either melanized or non-melanized, did not affect the transcription of the examined genes and the production of any tested cytokines suggesting that only the germ tubes and the hyphae are able to activate the monocytes (Figures S3 and S4 in Supplementary Material).

## Discussion

Considering that activation of the innate immune system has a crucial role in recognition and control of filamentous fungal infections (18), present study was carried out to obtain information about the response of monocytic cell line THP-1 to the dematiaceous fungus *C. lunata* and related species.

THP-1 monocytes were not able to phagocytose the large and melanized conidia of any tested *Curvularia* species. Despite the lack of attraction to the conidia, monocytes recognized *C. lunata*, but not *C. hawaiiensis* and *C. spicifera*, and preferably attached to the hyphae. In contrast to the *Curvularia* conidia, those of *A. fumigatus* were actively phagocytosed by the THP-1 cells in agreement with the results of previous studies (18). Different cell wall composition and highly distinct size of their conidia can explain this different affinity of the monocytes to the *Curvularia* and *Aspergillus* conidia. Although there are no available data about the response of monocytes to any similar dematiaceous mold, there are a few studies discussing the interaction of these fungi with other cell types. Recently, Reedy et al. (29) found that macrophages could not phagocytose the conidia of *Exserohilum rostratum*, another dematiaceous mold related to *Curvularia*. At the same time, macrophages also showed an attraction and attachment to the hyphae of *Exserohilum*. The different affinity of macrophages to the conidia and the hyphae was explained by the possibly different polysaccharide composition of the cell walls (29). Besides phagocytosis, only touching and dragging of the fungal cells, conidia and hyphal elements by macrophages to prevent the spread of the fungus into the different tissues was proven in cases of *A. fumigatus* and *C. albicans* (30).

Melanin is present in the cell wall of a wide range of fungal pathogens. Like Aspergilli, *Curvularia* species produce dihydroxynaphthalene-type melanin (DHN-melanin) (16). Melanin is generally considered as a virulence factor having role in the prevention of the immune recognition by masking the pathogen-associated molecular patterns on the surface of the conidia, as it was found in *A. fumigatus* (31–33). However, in our experiments, there was no difference in the response of monocytes to melanized and non-melanized conidia of *C. lunata* indicating that melanin content of the conidia has no significant effect on the recognition of this fungus by the THP-1 cells. Previously, comparison of killing rates of numerous dematiaceous yeasts by human neutrophils suggested that only melanization is not sufficient to assure the virulence (34).

MTT assay indicated that THP-1 cells did not damage significantly the *Curvularia* strains. Similarly, macrophages could not inhibit conidial germination and hyphal growth of *E. rostratum* (29). At the same time, THP-1 cells effectively decreased the viability of *A. fumigatus* during their interaction, as expected based on previous studies (20).

Transcription of immune-relevant genes of the monocytes during interaction with *C. lunata, C. hawaiiensis* and *C. spicifera* was examined by qRT-PCR analysis. A decrease in the transcription of the gene encoding CCR2, which is downregulated during monocytic differentiation (35), suggests the activation of THP-1 cells in response to *C. spicifera*. However, the unaltered or decreased expression of most tested genes, such as those encoding IL-6, IL-1β, CCR1, ITGAL, ITGAM, and ITGAX, suggests that *Curvularia* hyphae induce a moderate response in this cell type. After confrontation with *C. lunata*, transcription of HLA-DRA was significantly downregulated, assuming the lack of antigen presentation by monocytes despite of the attachment to the hyphae. In case of *A. fumigatus*, it was previously found that phagocytosis of the conidia did not induce the immediate expression of cytokine and chemokine genes in THP-1, which were activated only in the presence of the hyphae (18, 36). Similarly, we detected the induction of certain immune-relevant genes at 9 h postinoculation when the hyphae formation had already started.

In addition to the transcription analysis, production of certain cytokine proteins was measured after confrontation with the examined *Curvularia* strains. To the hyphae of *C. lunata*, THP-1 cells responded with intense IL-8 and IL-10 and moderate TNF-α production while they failed to produce IL-6. The chemokine IL-8 or CXCL8 is known to be involved in the immune response to fungal pathogens and it has a primary role in recruiting neutrophils to the site of infection (37, 38). IL-8 production and release by monocytes (including THP-1 cells) in response to confrontation with *A. fumigatus* is well documented by several studies (17, 18, 22). The low levels of the pro-inflammatory cytokines after interaction with *Curvularia* strains is somewhat surprising. These cytokines are involved in the effective immunity to fungal infections and their role has been studied and proven among others in response to *C. albicans, A. fumigatus*, and *C. neoformans* (37). In case of *C. lunata*, this situation can be partly explained by the high expression of the anti-inflammatory cytokine IL-10, which can repress pro-inflammatory responses and can inhibit the production of TNF-α, IL-6, and even IL-8 (39–41). In case of the THP-1 cells confronted with the *A. fumigatus* strain involved for comparison, this relatively high IL-10 production was not detected. Induction of IL-10 production in monocytes correlates well to that *Curvularia* strains and especially *C. lunata* can cause chronic infections even in immunocompetent persons (4) and are among the most frequent agents of allergic sinusitis and allergic bronchopulmonary disease (2, 4). It is known that certain pathogenic organisms possess mechanisms to modulate the immune response by enhancing IL-10 production and/or exploit the anti-inflammatory properties of IL-10 for their survival (42, 43). In the cases of *Histoplasma capsulatum* and *C. albicans*, the absence of IL-10 expression led to enhanced clearance of the fungi (44–46). Similarly, increased levels of serum IL-10 due to single nucleotide polymorphism in the *IL10* gene is associated with an increased susceptibility to *A. fumigatus* colonization and allergic bronchopulmonary aspergillosis (47). Despite the high IL-10 production, increased IL-8 level was measured after co-incubation with *C. lunata* that may suggest that neutrophils may have an important role in the immune response to this fungus as it was found in case of several other fungal species (33, 38). Interestingly, significantly increased relative transcript levels were measured for the genes encoding TNF-α and IL-10 in response to *C. spicifera* and *C. hawaiiensis* while secretion of these proteins by the monocytes could not be detected. Although the relative transcript level of *IL8* showed similar values as in case of *C. lunata*, there was no significant increase in protein release either. Considering that secretion of IL-10 and IL-8 is regulated primarily at a transcriptional level (48, 49), this is a surprising phenomenon. TNF-α production is regulated at a transcriptional and translational level as well (50, 51). Immune cells can display different attraction even to closely related fungal species. For example, different recognition of *C. albicans* and *C. glabrata* was observed by Netea et al. (52) and a distinct IL-1β release and ROS production of macrophages induced by *C. albicans* and *C. parapsilosis* (53). *Aspergillus* species (i.e., *A. fumigatus, A. flavus, A. niger*, and *A. terreus*) also induced significantly different release of IL-6 and TNF-α by human monocytes (54).

As a conclusion, our results show that THP-1 monocytes cannot effectively phagocytose the conidia of *Curvularia* species and there is a clear difference in response of these cells to the three investigated strains. They attach to the hyphae and respond to those of *C. lunata* by IL-10 and IL-8 production. Further studies to clarify the mechanisms in the background of the enhanced IL-10 production and the possible association of this phenomenon with the chronic mycoses caused by this fungus are needed. Considering the upregulation of IL-8, the role of neutrophils in the clearance of *Curvularia* infections is also worth to examine.

## Author Contributions

ET carried out most of the experimental work, performed the statistical analysis, and participated in the evaluation of the results and drafting the manuscript. ÉB, AH, CS, and MH performed experiments, analyzed data, and participated in the writing of the manuscript. GN performed experiments and participated in the evaluation of the results. IN, CV, and TP designed and coordinated the study and participated in the writing and the edition of the manuscript.

## Conflict of Interest Statement

The authors declare that the research was conducted in the absence of any commercial or financial relationships that could be construed as a potential conflict of interest.
