# Supplementary material for: Interaction of THP-1 Monocytes with Conidia and Hyphae of Different Curvularia Strains
Source: Front Immunol. 2017 Oct 18;8:1369. doi: 10.3389/fimmu.2017.01369 (PMC5651265; doi:10.3389/fimmu.2017.01369)
Supplement: Supplementary file 1 [file presentation_1.pdf]

Supplementary material

Interaction of THP-1 monocytes with conidia and hyphae of different *Curvularia* strains

Eszter Judit Tóth, Éva Boros, Alexandra Hoffmann, Csilla Szebenyi, Mónika Homa, Gábor Nagy, Csaba Vágvölgyi, István Nagy, Tamás Papp

Corresponding author: Tamás Papp
E-mail: pappt@bio.u-szeged.hu
Phone: (+36) 62 544516, Fax: (+36) 62 544823

Supplementary Figure S1

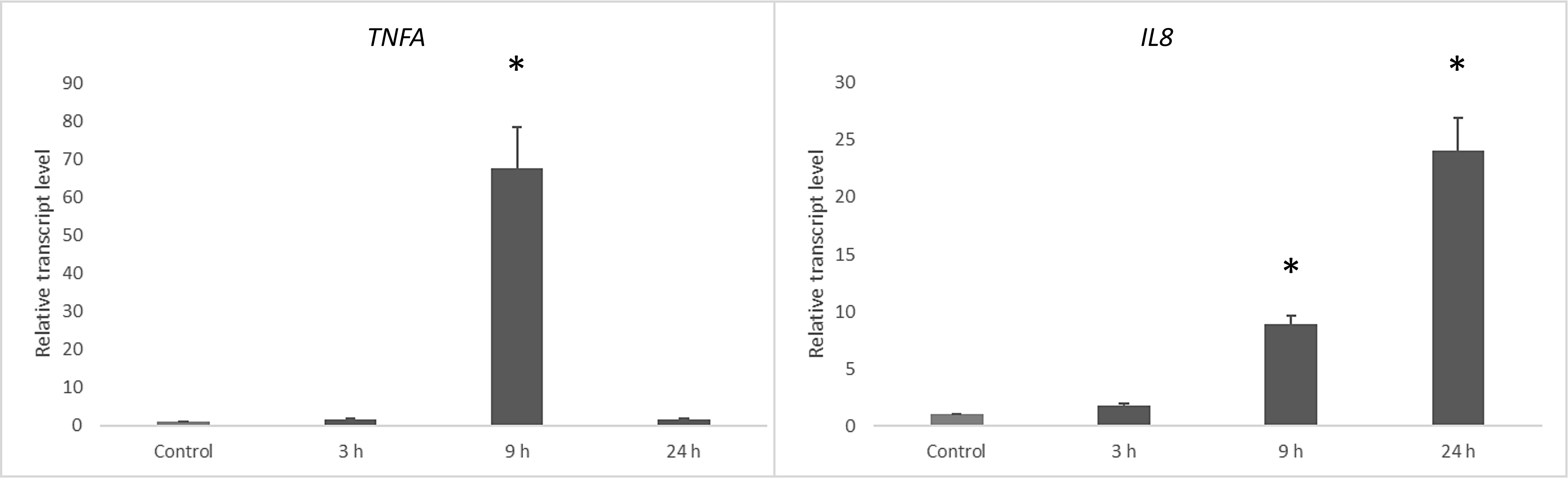

FIGURE S1: Relative transcript levels of *TNFA* and *IL8* in THP-1 monocytes after interaction with *A. fumigatus*. Presented values are averages of three independent experiments. Relative transcript values followed by * significantly differed from the 0 h control (taken as 1), according to the paired t-test ($p < 0.05$). Transcription levels of *TNFA* and *IL8* were found to be much higher than those induced by *C. lunata* (see Fig. 4).

Supplementary Figure S2

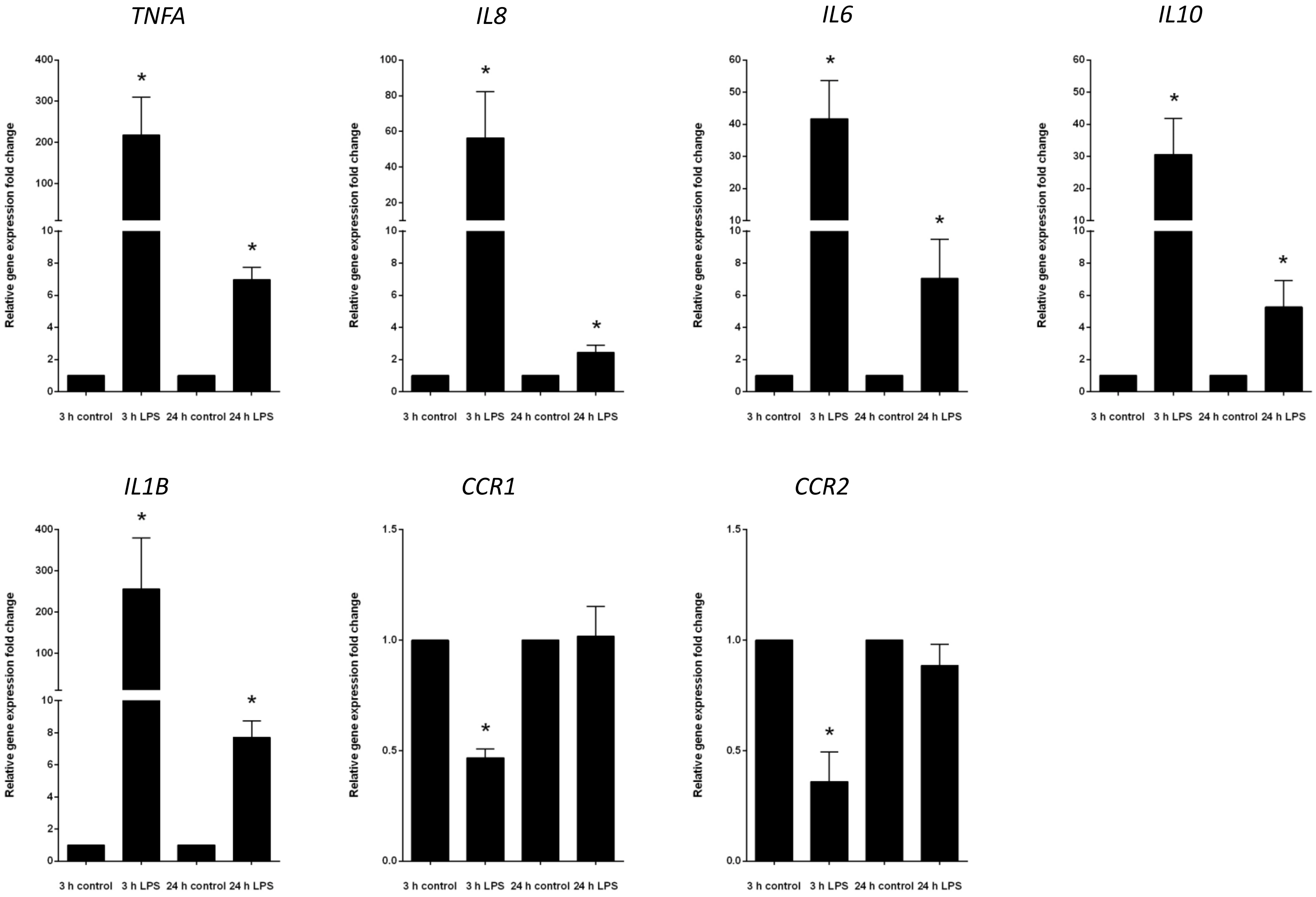

FIGURE S2: Relative transcript levels of *TNFA*, *IL8*, *IL6*, *IL10*, *IL1B*, *CCR1* and *CCR2* in THP-1 monocytes after induction with LPS for 3 and 24 h. Presented values are averages of three independent experiments; error bars represent standard deviations. Relative transcript values followed by * significantly differed from the control (taken as 1), according to the paired t-test ($p < 0.05$).

Supplementary Figure S3

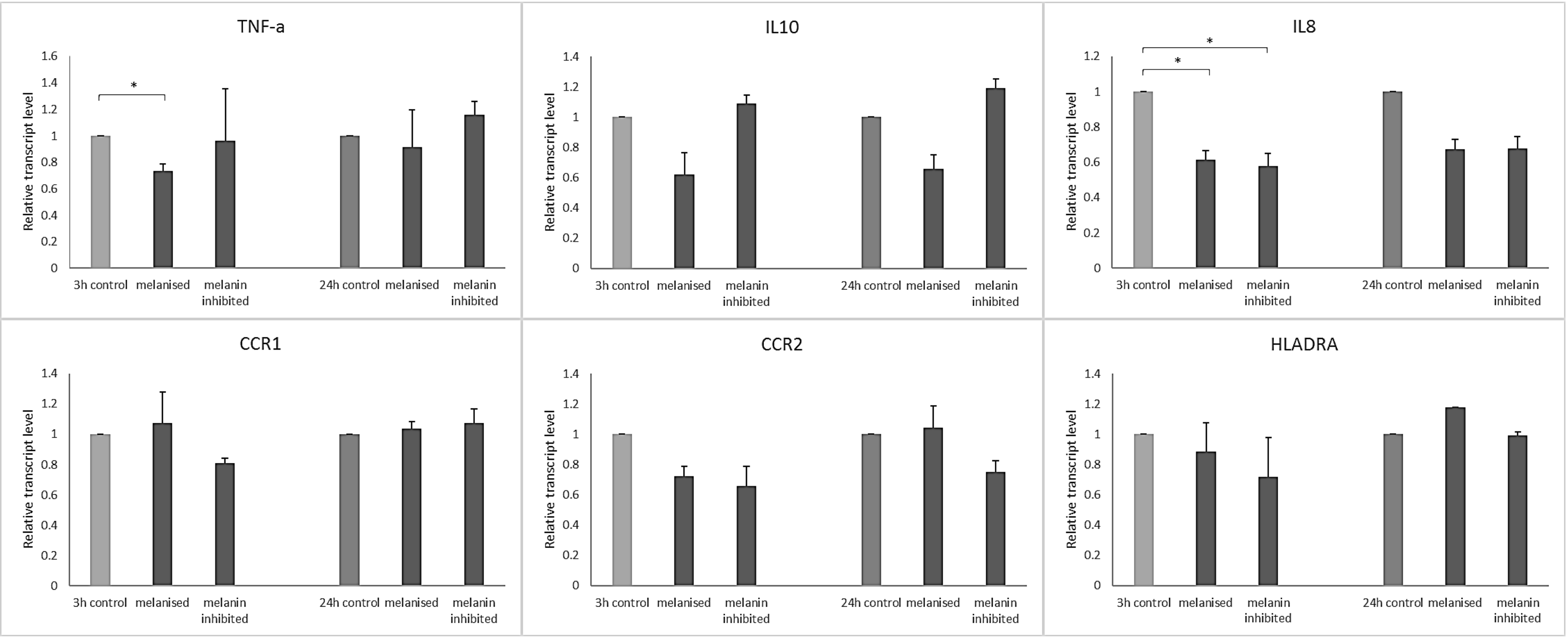

FIGURE S3: Relative transcription of immune relevant genes in THP-1 monocytes after 3 and 24 hours of co-incubation with melanized and melanin inhibited (heat inactivated) conidia of *C. lunata*. Presented values are averages of three independent experiments; error bars represent standard deviations. Relative transcript values followed by * significantly differed from the control (taken as 1), according to the paired t-test ($p < 0.05$).

Supplementary Figure S4

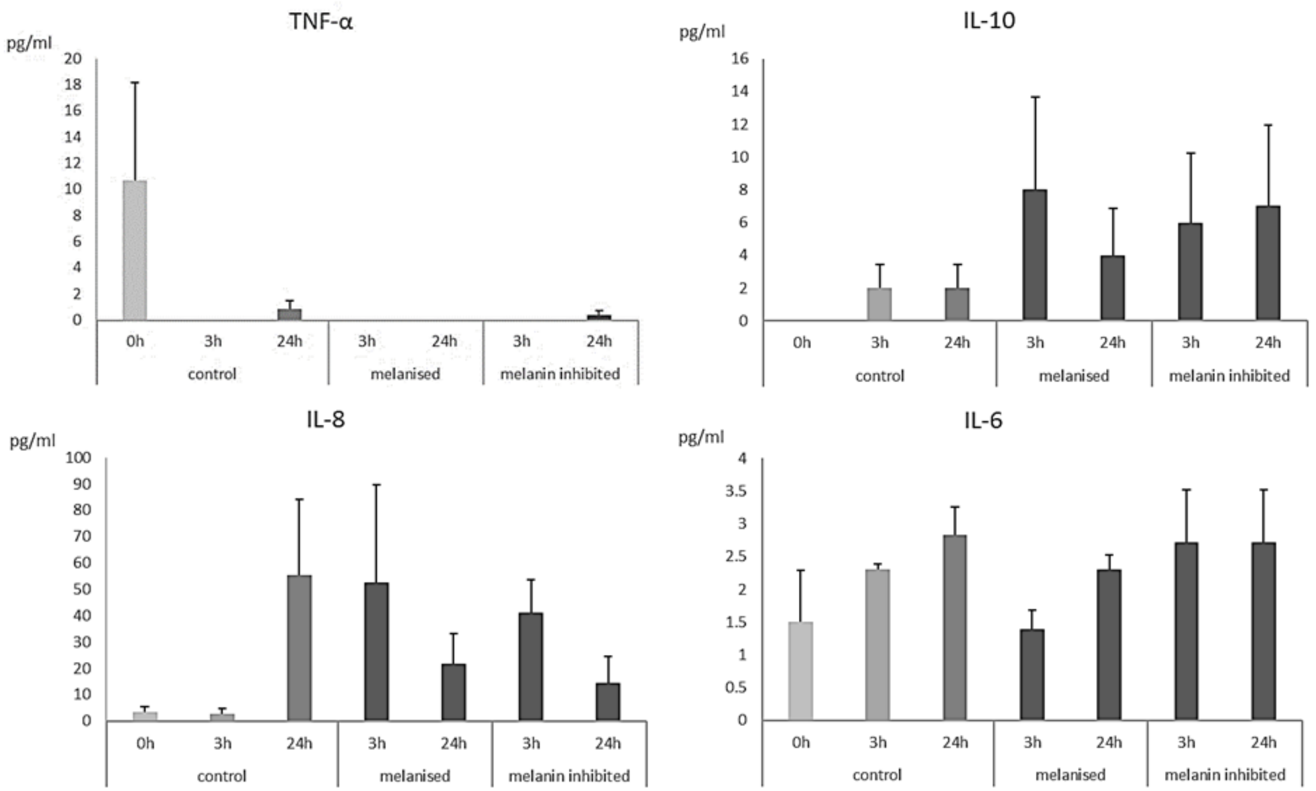

FIGURE S4: Production of cytokines TNF-α, IL-10, IL-8 and IL-6 of THP-1 monocytes within response to melanized and melanin inhibited (heat inactivated) conidia of *C. lunata* after 3 and 24 hours of co-incubation. Results are presented as averages of three independent experiments; error bars represent standard deviations.
